# Achieving sustained extrauterine life: Challenges of an artificial placenta in fetal pigs as a model of the preterm human fetus

**DOI:** 10.14814/phy2.14742

**Published:** 2021-03-02

**Authors:** Alex J. Charest‐Pekeski, Ayman Sheta, Luiza Taniguchi, Mark J. McVey, Alejandro Floh, Liqun Sun, Tanroop Aujla, Steven K. S. Cho, Jiaqi Ren, Lynn Crawford‐Lean, Celeste Foreman, Jessie Mei Lim, Brahmdeep S. Saini, Marvin Estrada, Anson Lam, Jaques Belik, Dariusz Mroczek, Megan Quinn, Stacey L. Holman, Jack R. T. Darby, Mike Seed, Janna L. Morrison, Christoph Haller

**Affiliations:** ^1^ Department of Physiology University of Toronto Toronto Ontario Canada; ^2^ Translational Medicine The Hospital for Sick Children Toronto Ontario Canada; ^3^ Department of Pediatrics Division of Neonatology The Hospital for Sick Children Toronto Ontario Canada; ^4^ Division of Cardiology The Labatt Family Heart Centre The Hospital for Sick Children Toronto Ontario Canada; ^5^ Department of Anesthesia and Pain Medicine Department of Anesthesiology and Pain Medicine The Hospital for Sick Children University of Toronto Toronto Ontario Canada; ^6^ Department of Physics Ryerson University Toronto Ontario Canada; ^7^ Department of Critical Care Medicine The Hospital for Sick Children Toronto Ontario Canada; ^8^ Early Origins of Adult Health Research Group Health and Biomedical Innovation Clinical and Health Sciences University of South Australia Adelaide South Australia Australia; ^9^ Division of Cardiovascular Surgery The Labatt Family Heart Centre The Hospital for Sick Children University of Toronto Toronto Canada; ^10^ Institute of Medical Science University of Toronto Toronto Ontario Canada; ^11^ Lab Animal Services Research Institute The Hospital for Sick Children Toronto Ontario Canada

**Keywords:** artificial placenta, cannulation, heart failure, preterm pig model

## Abstract

Artificial placenta (AP) technology aims to maintain fetal circulation, while promoting the physiologic development of organs. Recent reports of experiments performed in sheep indicate the intrauterine environment can be recreated through the cannulation of umbilical vessels, replacement of the placenta with a low‐resistance membrane oxygenator, and incubation of the fetus in fluid. However, it remains to be seen whether animal fetuses similar in size to the extremely preterm human infant that have been proposed as a potential target for this technology can be supported in this way. Preterm Yucatan miniature piglets are similar in size to extremely preterm human infants and share similar umbilical cord anatomy, raising the possibility to serve as a good model to investigate the AP. To characterize fetal cardiovascular physiology, the carotid artery (*n* = 24) was cannulated in utero and umbilical vein (UV) and umbilical artery were sampled. Fetal UV flow was measured by MRI (*n* = 16). Piglets were delivered at 98 ± 4 days gestation (term = 115 days), cannulated, and supported on the AP (*n* = 12) for 684 ± 228 min (range 195–3077 min). UV flow was subphysiologic (*p* = .002), while heart rate was elevated on the AP compared with in utero controls (*p* = .0007). We observed an inverse relationship between heart rate and UV flow (*r* ^2 ^= .4527; *p* < .001) with progressive right ventricular enlargement that was associated with reduced contractility and ultimately hydrops and circulatory collapse. We attribute this to excessive afterload imposed by supraphysiologic circuit resistance and augmented sympathetic activity. We conclude that short‐term support of the preterm piglet on the AP is feasible, although we have not been able to attain normal fetal physiology. In the future, we propose to investigate the feasibility of an AP circuit that incorporates a centrifugal pump in our miniature pig model.

## Graphical abstract

The artificial placenta (AP) as an extrauterine environment to preserve fetal physiology and to promote fetal maturation forms a promising concept to reduce morbidity and mortality in the extremely premature. We have achieved successful transition of fetal minipigs on the AP but have encountered heart failure and fetal demise within several hours of support. Our experience highlights modes of failure and ways to improve AP technology to advance this approach to clinical application.

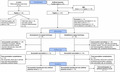

## 1 INTRODUCTION

Preterm birth, defined as delivery before 37 weeks gestation, is a major global health problem (Beck et al., 2010). The World Health Organization estimates the current worldwide incidence of preterm birth is approximately 15 million preterm deliveries per year, with the rate of preterm birth on the rise (Blencowe et al., 2013). While the widespread creation of dedicated teams and facilities for caring for preterm infants has resulted in tremendous advances in the outcomes of these patients, morbidity and mortality remain high in the extremely premature (<28 weeks gestation), particularly in those born close to the lower limit of viability (22 weeks gestation) (Stoll, 2015). At this gestation, the lungs are in the late canalicular and early saccular phases of development (Glass et al., 2015), and lack the diffusion capacity to deliver adequate gas exchange to support the newborns’ oxygen requirements (Coalson, 2003). Support of pulmonary function with mechanical ventilation has proven harmful to the immature lungs, causing pulmonary developmental arrest and irreversible lung damage secondary to barotrauma and oxygen toxicity (Carraro et al., 2013). Antenatal corticosteroids and surfactant replacement therapy have significantly improved survival, but have not led to a reduction in the incidence of bronchopulmonary dysplasia, highlighting the need for alternative solutions for overcoming respiratory failure and irreversible lung injury in preterm infants (Carraro et al., 2013; Coalson, 2003).

The concept of an artificial placenta (AP), whereby gas exchange is achieved using an extracorporeal membrane oxygenator (ECMO) connected to the fetal circulation via the umbilical vessels has been investigated since the 1950s (Westin et al., 1958). Recent research has established the feasibility of AP support of preterm sheep for periods of up to a month using a pumpless ECMO circuit (Arens et al., 2011; Church, Coughlin, et al., 2018; Church, Perkins, et al., 2018; Church, Werner, et al., 2018; El‐Sabbagh et al., 2018; Gray et al., 2013; Hornick et al., 2018; Lawrence et al., 2018; Miura et al., 2012, 2015, 2016; Partridge, Davey, Hornick, McGovern, et al., 2017; Reoma et al., 2009; Rochow et al., 2013; Schoberer et al., 2014; Usuda et al., 2017, 2019; Westin et al., 1958; Zapol et al., 1969). Importantly, two groups have provided evidence of near‐normal fetal growth and development using this approach without any of the typical organ injury associated with preterm birth (Hornick et al., 2018; Partridge, Davey, Hornick, McGovern, et al., 2017; Usuda et al., 2017, 2019).

Despite these successes, there are some limitations of the preterm sheep as a model for the extremely preterm human infant. Importantly, extremely preterm human fetuses during their canalicular phase of lung development are 500–750 g (Kiserud et al., 2018), while the smallest sheep used in AP studies are twice that size (~1.0 kg) (Arens et al., 2011; Church, Coughlin, et al., 2018; Church, Perkins, et al., 2018; Church, Werner, et al., 2018; El‐Sabbagh et al., 2018; Gray et al., 2013; Hornick et al., 2018; Lawrence et al., 2018; Miura et al., 2012, 2015, 2016; Partridge, Davey, Hornick, McGovern, et al., 2017; Reoma et al., 2009; Rochow et al., 2013; Schoberer et al., 2014; Usuda et al., 2017; Westin et al., 1958; Zapol et al., 1969). Fetal size is associated with hemodynamic factors such as umbilical vessel caliber and blood pressure that are important for ECMO support (Rafat & Schaible, 2019; Tanaka et al., 2019). Furthermore, the vascular anatomy of the sheep umbilical cord is different from that of humans, with sheep having two umbilical arteries and two umbilical veins in contrast to human fetuses that have only one umbilical vein (UV) (Benirschke & Kaufmann, 2000; Steven, 1968). This allows for cannulation and connection of one umbilical artery (UA) and one UV onto AP support in sheep, while the other pair of umbilical vessels remains attached to the placenta allowing fetal oxygenation from the mother. This helps to limit the interruption of oxygen delivery to the fetus, permitting a controlled transition of the fetal sheep onto AP support, which may not be as easily achieved in humans. In contrast to sheep, fetal Yucatan miniature pigs have the same number of umbilical arteries and veins as humans. With respect to the umbilical cord length, the porcine umbilical cord is shorter than the human cord and is similar in length to the sheep cord. Additionally, the pig has a diffuse epitheliochorial placenta, whereas, sheep and humans have a cotyledonary epitheliochorial and a discoid hemochorial placenta, respectively. Despite these structural differences, the placentas of all species appear to have similar functions and hemodynamic characteristics (King, 1992). While the sheep and human brain both develop in late gestation; the sheep brain is more mature than the human brain (Morrison, 2018). At 101 days gestation, the miniature pig brain is at a similar stage of maturity as in preterm humans (Dobbing & Sands, 1979; Eiby et al., 2013) and the average body weight of approximately 650 g corresponds to a human newborn delivered at 23–25 weeks gestation (500–750 g) (Kiserud et al., 2018). The aim of the current study was, therefore, to investigate the feasibility of providing AP support to preterm miniature pigs using approaches recently pioneered in fetal sheep, thereby potentially bringing this exciting technology closer to human translation.

## 2 METHODS

The current study consisted of two groups. First, animals were studied in utero in order to attain accurate reference data regarding normal pig circulatory physiology and hemodynamics. This group functioned as the control/comparative group to the second group of animals that were supported on the AP.

### 2.1 Animal treatment and ethical standards

Fetal in utero studies were performed in five Large White Landrace Cross sows from the Roseworthy Piggery (University of Adelaide) at the Preclinical Imaging and Research Laboratories, South Australian Health and Medical Research Institute (SAHMRI) with approval from the SAHMRI Animal Ethics Committee for the collection of blood gases, electrolytes, lactate, glucose, blood pressure, and heart rate (HR) data. All other experiments were performed at The Hospital for Sick Children, Research Institute, Peter Gilgan Center for Research and Learning, Lab Animal Services in Toronto, Ontario with approval from the SickKids Animal Care Committee. All surgical procedures were in compliance with the Canadian Council on Animal Care, Ontario Ministry of Agriculture, Food and Rural Affairs, Animals for Research Act guidelines, and Australian Code for the Care and Use of Animals for Scientific Purposes. All research staff understood and complied with the ARRIVE guidelines (Kilkenny et al., 2010). Pregnant Yucatan sows (*n* = 6, Memorial University of Newfoundland, Animal Care Services; *n* = 21, Sinclair BioResources) were transported in line with the terms of the Health of Animals Act of Canada. Sows were housed for a minimum of 1 week prior to the experiment to allow acclimatization to human interaction and to the laboratory environment. They were provided with *ad libitum* access to food, water, and environmental enhancements as per local vivarium standard operating procedures.

### 2.2 Fetal in utero blood gas, electrolytes, lactate, and glucose status

Large White Landrace Cross Gilts (*n* = 5) at 91, 98, and 106 days gestation (term = 115 days) were anesthetized with the inhalation of isoflurane via a face mask and intramuscular (IM) ketamine injection (20 mg/kg), intubated, and maintained on isoflurane (Fluothane, ICI), with 2 L O_2_ and 4 L/min air. Gilts were placed on their back on the surgical table and an incision was made on each side of the abdomen to expose the uterus, which was incised to expose each fetus. In 24 piglets, blood samples were collected from the UV for the measurement of whole blood partial pressure of oxygen (PO_2_), partial pressure of carbon dioxide (PCO_2_), pH, base excess (BE), oxygen saturations (SO_2_), hematocrit (Hct), and hemoglobin (Hgb) with a blood gas analyzer (RAPIDPoint 500 – Siemens Healthineers) and temperature corrected to 39°C. In six piglets, a catheter was placed in the fetal carotid artery and connected to a PowerLab data acquisition system via a displacement transducer and quad‐bridge amplifier (ADInstruments). Blood pressure and HR were measured and sampled at a rate of 1,000 Hz, digitized and recorded using LabChart 8 (ADInstruments). Blood pressure and HR data were exported from LabChart 8 (ADInstruments) in consecutive 30‐s epochs.

### 2.3 Sow anesthesia and preparation for AP experiments

General anesthesia was induced (10 mg/kg ketamine, 0.20 mg/kg acepromazine, and 0.015 mg/kg atropine sulfate; IM; CDMV, Saint‐Hyacinthe) in sows to facilitate intubation with a #9 cuffed endotracheal tube (Portex^®^ Soft Seal^®^ Tracheal Tube; Smiths Medical Ltd). General anesthesia was maintained in mechanically ventilated (100% F_i_O_2_, 6 L/min FGF, 15 ml/kg V_t_, and 5 cm H_2_O PEEP) sows with the inhalation of 2%–4% isoflurane (Fresenius Kabi). Anesthetized sows were positioned in the left lateral position to minimize aortocaval compression. The operating room was heated to 23–25°C and sows were draped to maintain normothermia. An 18‐gauge ear vein intravenous (IV) cannula (Becton, Dickinson and Company) was inserted to deliver 100 ml/h of 0.9% normal saline (NS) (Baxter Inc.).

### 2.4 Determination of UV blood flow using MRI

Pregnant sows (*n* = 4; gestational age 107 ± 3 days) were scanned 2 days prior to the AP experiment, using a 3 Tesla clinical magnetic resonance imaging (MRI) system (Siemens Prismafit) to allow the quantification of normal in utero UV flow according to our previously published technique (Duan et al., 2019; Seed et al., 2012). Prior to all MRI scans, Loperamide (30–35 ml; PO) was given (~45 min) to overcome movement artifacts during the scanning period.

#### 2.4.1 3D volumetry

**FIGURE 1:**
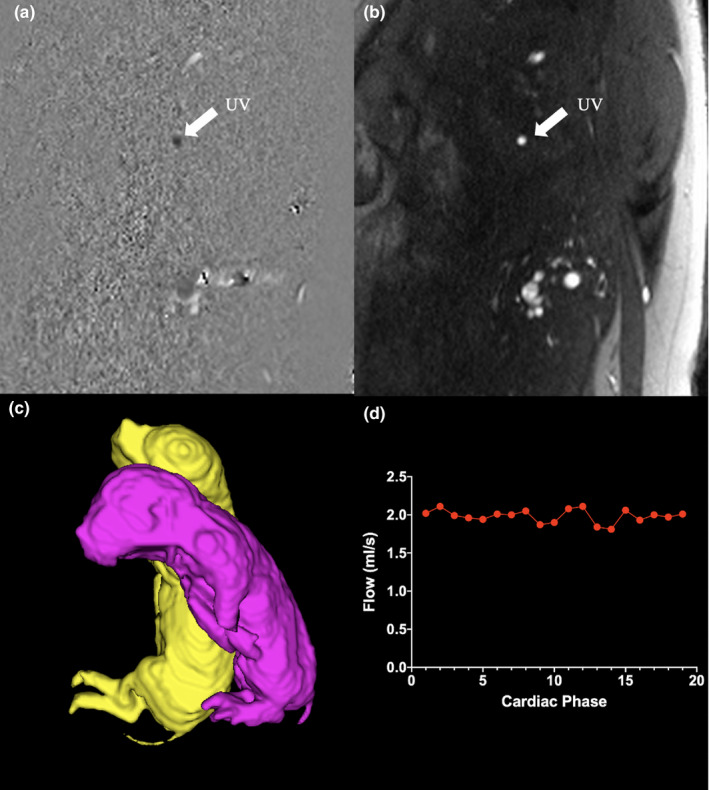
Measurement of umbilical blood flow in utero by cine phase‐contrast MRI. Phase (a) and modulus (b) images from the cine phase‐contrast acquisition, segmentation of fetal volumes for the quantification of fetal weight (c), and typical non‐pulsatile flow profile obtained from the umbilical vein (d)

Using a 3‐dimensional steady‐state free precession acquisition of the uterine cavity, fetal volumetric images were acquired (TE =1.36 ms, slice thickness =2 mm, field of view =120 mm, matrix size =272 × 272, number of signal averages = 1; Figure 1). Collected images were post‐processed using a free segmentation software, *ITK*‐*Snap* (itk‐SNAP), and manually segmented for each fetus (Yushkevich et al., 2006). Fetal weight was estimated from total body volume using a previously described conversion factor (Baker et al., 1994) and was used to normalized acquired blood flow data.

$$\text{Fetal}\;\text{Weight}\;\left(\text{kg}\right)=\frac{\text{Fetal}\;\text{Volume}\;\left(\text{ml}\right)\times 1.03+120\;\text{ml}}{1000}$$

#### 2.4.2 Phase‐contrast MRI for blood flow quantification

Cine phase‐contrast MRI acquisitions targeting the intrahepatic UV were performed using metric optimized gating according to our previously published technique (flip angle =30°, slice thickness = 5 mm, field of view = 240 mm, in‐plane resolution = 1.0 × 1.0, number of signal averages = 3, TE = 3.18 ms, TR = 7 ms; Figure 1). The PC‐MRI acquisitions were performed using a velocity‐encoding vector of 50 cm/s (*n* = 16) (Duan et al., 2019; Seed et al., 2012). Images were then post‐processed using Segment v3.0 R7732 (Medviso AB) where contours were semi‐automatically placed around the vessel of interest and propagated through all phases of the cardiac cycle to generate flow data.

### 2.5 Surgical protocol for AP experiments

Approximately 200–300 ml of citrated whole blood was collected from anesthetized sows that were cannulated via the posterior tibial artery (*n* = 24) or carotid artery (*n* = 3) to obtain maternal donor blood for fetal transfusion resuscitation and priming of the extracorporeal circuit for AP experiments. Sows were given an empiric IV bolus of 100 units/kg unfractionated heparin (UFH; Fresenius Kabi) every 2 h via ear vein cannula to prevent thromboses. For the initial 18/27 experiments, 0.02 mg/kg buprenorphine (Ceva Animal Health Inc.) was delivered to the sow 10 min prior to the incision for the cesarean section, whereas for the subsequent 9/27 experiments this was replaced with pre‐incisional infiltration with subcutaneous 7 mg/kg 2% lidocaine containing 1:200,000 epinephrine (AstraZeneca Inc.). After delivery of the fetal pigs, anesthetized sows were euthanized with 106 mg/kg Euthanyl (Pentobarbital sodium, Bimeda Animal Health Inc.).

**FIGURE 2:**
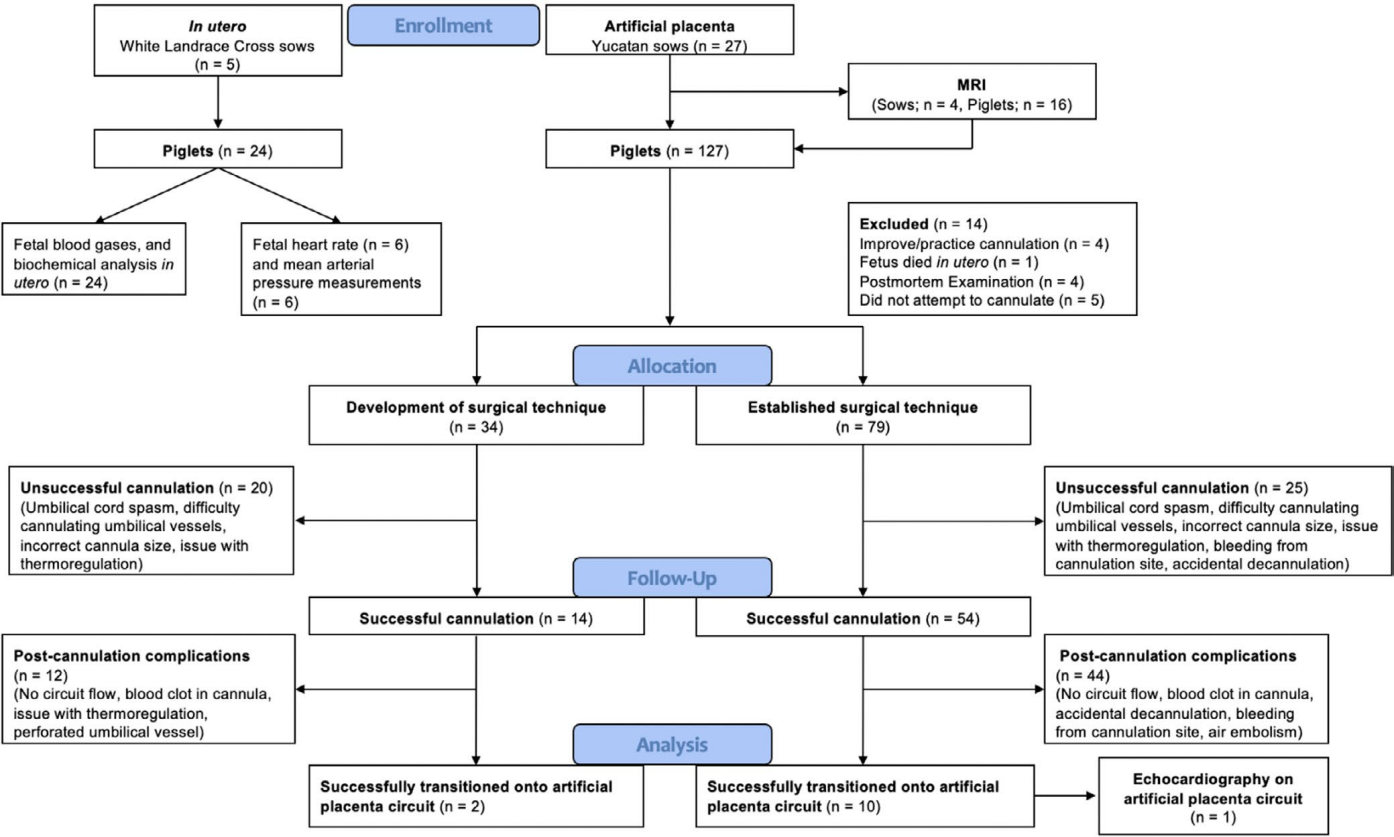
Consort diagram of in utero fetal blood gases, and biochemical analysis and the developed and established surgical technique for the AP experiments. Fetal piglets that were unsuccessfully cannulated or those that were successfully cannulated but had post‐cannulation complications were euthanized

A total of 27 sows, 101 ± 6 days (*n* = 24) gestation (term = 115 days) were used in this study with an average litter size of 4.5 ± 1.9 fetuses (Figure 2). In total, there were 127 piglets in our current study with a mean body weight of 651 ± 240 g (*n* = 107). We experienced a significant “learning curve” with establishing fetuses onto the system. We successfully cannulated 68 fetal pigs; however, 56 were euthanized due to post‐cannulation complications (no circuit flow, *n* = 35; air embolism in circuit, *n* = 5; accidental decannulation, *n* = 5; perforated UV, *n* = 4; umbilical vessel thrombosis, *n* = 3; bleeding from cannula, *n* = 3; failure to maintain body temperature when moved into BioBag, *n* = 1). Fetal pigs that could not be transitioned to AP support or those that were not successfully cannulated, were euthanized with 1.2 ml/kg of Euthanyl. In this study, 12 fetal pigs were successfully transitioned to and supported on our AP circuit. A successful trial was defined as >3 h of AP support. We achieved a successful trial in 10 of our last 19 experiments. A flow diagram of our results is shown in Figure 2.

#### 2.5.1 Preparation of the fetus for cannulation

After appropriate positioning of the sow, hair in the surgical field was clipped and the incision site marked. An abdominal incision was made, and the uterus was exposed. After locating a fetal head, the uterus was incised, and the fetus delivered in such a way as to prevent compromise of the cord by stretching or kinking. Fetuses were given a single IM injection of 2.5 mg (5 mg/kg) ketamine and 0.5 mg (1 mg/kg) rocuronium (Sandoz Inc.). Fetal normothermia was maintained by continuously bathing the umbilical cord and fetus with heated 0.9% NS (Baxter Inc.) at 37–40°C. If needed, significant spiraling of the cord was corrected by the repositioning of the fetus to achieve a relatively parallel umbilical vessel alignment. We initially pretreated the umbilical cord with 100 mg/kg of topical papaverine and/or 2 mg/kg of papaverine (Sandoz Inc.) injected into the perivascular space to prevent periprocedural vasospasm of the umbilical vessels (piglets: *n* = 66/127). This approach was subsequently changed to the topical application of 100 mg/kg papaverine and combined administration of 50 units/kg/h UFH and 0.1 mg/kg/min papaverine via the AP circuit.

A major aspect of our initial experience was the development of a cannulation technique that addressed three key deliverables for the successful transition to the AP circuit: (1) minimal negative effects on fetal hemodynamics during cannulation; (2) minimal trauma to the umbilical cord while maximizing cannula size; and (3) a low complexity, minimal touch technique to improve reproducibility, and speed. We have retrospectively attributed 34 piglets to this arm of our experimental experience (Figure 2).

#### 2.5.2 Approach to the vessels

Initial cannulation trials were performed after ligation and division of the umbilical cord. Although this disrupted umbilical blood flow completely, positioning the fetus on a separate heating pad and aligning the umbilical cord with the circuit were attractive for ease of cannulation to improve the timely transition to the AP. Maintenance of placental blood flow; however, was considered preferable to minimize fetal distress and to reduce the incidence of cord spasm. We adapted our technique to keep the exteriorized fetus on the sow's abdominal wall with the umbilical cord exposed but relaxed. To maintain umbilical blood flow for as long as possible, we initially decided to cannulate one UA first, followed by the UV, with subsequent connection and initiation of flow to the AP circuit prior to the cannulation of the second UA. This increased stability during cannulation, but the ongoing placental flow, while on partial AP support led to a net fetal volume loss. We, therefore, decided to perform both arterial cannulations first, followed by the venous cannulation and initiation of AP flow. This continued to be our preferred strategy for the remainder of the experiments.

In the early days of our experience, access to umbilical vessels was achieved by careful surgical dissection of the Wharton's jelly to expose each vessel wall. A small Mixter clamp was used to loop 2–0 Silk ties around each vessel to secure the cannulas after placement. The vessels were then partially incised with a #11 scalpel and cannulas were introduced. Once in place, silk ties were tied down on each cannula. This approach necessitated excess surgical manipulation and quite frequently led to vasospasm of both vein and arteries. Introduction of cannulas with sizes close to the inner diameter of the vessel was further complicated by the fragile vessel wall as it tore easily or did not allow the cannula to advance appropriately. We addressed these challenges by a hybrid technique combining vessel incision with Seldinger‐type introduction of a plastic stent mounted on a tapered introducer. Once the stent was in place, the introducer was exchanged with a size‐matched cannula that could then be positioned and repositioned within the cord. This was a significant improvement with the reduction of vessel trauma and higher cannula to vessel size ratio. Disadvantages were the relative complexity and length of the procedure, extensive manipulation of the cord, as well as blood loss during cannula insertion.

#### 2.5.3 Selection of cannulas

In the early stages of our experiments, clinically approved pediatric arterial and venous cannulas of various sizes were used. However, available cannula sizes were inappropriate for smaller fetuses and had unfavorable wall thickness to luminal diameter ratio. Cannula length and priming volume were also disadvantageous for these small size piglets. Alternative catheters from both clinical and veterinary suppliers could not address these deficits. We, therefore, developed a series of short custom‐made polymer cannulas ranging from 2.1 to 3.3 mm in diameter with low wall thickness to luminal diameter ratio. Similar to percutaneous IV catheters, the cannulas featured a beveled steel needle within a plastic obturator size‐matched to the respective luminal diameter of the cannula. A female Luer connection at the circuit side of the cannula allowed for quick connection with the circuit. Fitting non‐screw plugs allowed us to temporarily occlude the cannula and prevent blood spillage after placement. Silk sutures used to secure the cannulas to the vessels were placed by carefully guiding the needle through the Wharton's jelly encircling each of the vessels separately. These technical modifications allowed us to insert large bore cannulas directly into the umbilical vessels, with minimal handling and no preceding surgical preparation. Cannulation times as short as two minutes to the initiation of AP flow could be achieved (Table 1).

**TABLE 1:** Summary of the 12 fetal piglets that were successfully transitioned onto AP support

Piglet number	Fetal weight (g)	Time on AP support (min)	Duration of cannulation (min)
7	600	217	2
11	1,500	433	NA
14	NA	369	3
15	NA	368	NA
17	NA	477	2
19	627	369	2
20	612	307	5
21	NA	3077	2
22	NA	548	1
24	826	936	3
25	535	917	5
27	500	195	2
Mean ± SD	743 ± 350 (*n* = 7)	684 ± 790	2.7 ± 1.3 (*n* = 10)

Abbreviations: NA, not available; SD, standard deviation.

Despite these significant improvements, we intermittently continued to experience cannulation related problems that were mainly attributed to cord spasm localized at the cannula tip, oversizing of the cannula with resulting vessel trauma, dissection, and occasional manufacturing defects.

### 2.6 AP circuit

Throughout the course of our AP experiments, we used three different hollow fibers, low resistance membrane oxygenators: Quadrox‐I neonatal, Quadrox‐ID pediatric (Maquet), and a rabbit oxygenator (Xijian Medical) with priming volumes of 38, 80, and 15 ml of heparinized maternal blood, respectively, and ~20 ml of additional crystalloid prime in the tubing. Oxygenators consisted of an arterial (inflow) and venous (outflow) port, connected to 3/16″ Tygon PVC tubing with P.h.i.s.i.o coating (LivaNova PLC). PlasmaLyte (Baxter Inc.) was used to prime the oxygenator and tubing prior to adding maternal blood. Blood flow from the UA entered the oxygenator and returned to the fetal heart via the UV. The majority (*n* = 11/12) of our successful AP trials were entirely pumpless in design. However, on a single, successful trial, a roller pump (Masterflex Easy‐Load; Cole‐Parmer Instrument Company LLC.) was used intermittently at a low speed, to help establish circuit flows.

### 2.7 Artificial uterine environment

**FIGURE 3:**
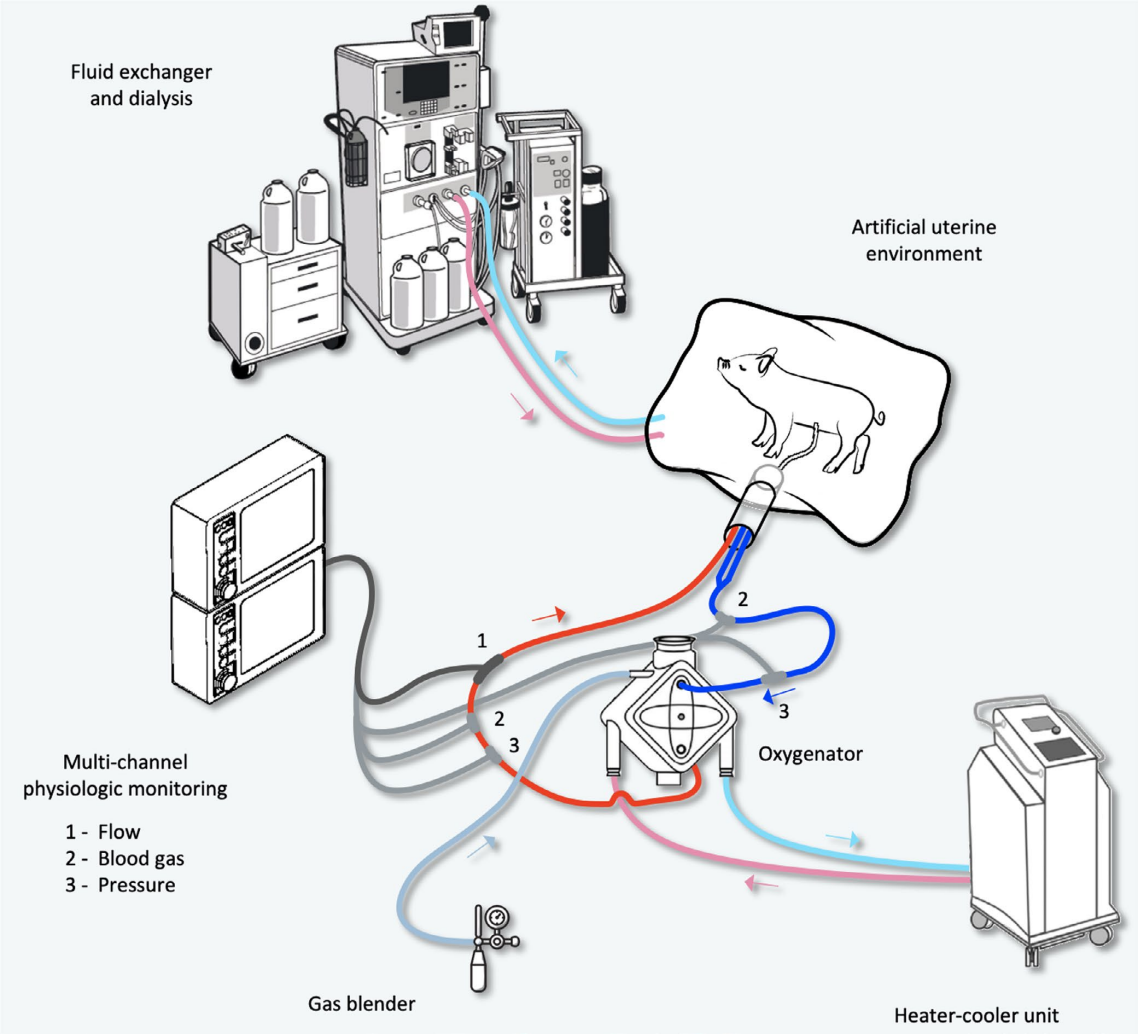
Equipment layout and circuit of the AP set‐up. Fetal pigs were maintained in an artificial uterine environment consisting of polyurethane BioBag filled with heated LR. LR was intermittently exchanged and continuously circulated in a large fluid reservoir. Blood was pumped by the fetal heart through the UAs through a neonatal or pediatric ECMO oxygenator (e.g., Maquet Quadrox‐ID pediatric), connected to a heater‐cooler unit and a gas blender and low flow meter supplied with a mixture of nitrogen, oxygen, and medical air. A Transonic HXL flow sensor was used for the physiologic monitoring of blood flow

Fetal piglets that were successfully cannulated were transitioned onto AP support and maintained in a custom‐made polyurethane film BioBag. The BioBag incorporated inflow and outflow ports for the circulation of NS (*n* = 15/27, initial experiments) or Lactated Ringer's (LR) (*n* = 12/27, later experiments) (Baxter Inc.) and periodic exchange (approximately every 10 h of AP support) of fluid in longer trial runs. Saline or LR were warmed to 39 ± 1°C, in a large reservoir and circulated by a heat therapy pump (HTP‐1500; Adroit Medical Systems Inc.) through the BioBag at a rate of 0.9 L/min. To maintain the temperature of the saline or LR solution in the BioBag, a radiant heater (Ohio^®^; Ohmeda Medical Inc.) and contact heat pad (Maxitherm^®^; Weiss Technik North America Inc.) were placed above and below the BioBag, respectively. The BioBag was covered with a drape during the majority of the experiments to maintain a dark environment. A Mikro‐Tip^®^ temperature probe (Millar Inc.) was placed underneath the BioBag to monitor temperature throughout the course of the experiments. In our later experiments, with improvements to the design of the BioBag, the temperature probe was inserted into the BioBag with the aim of improving the accuracy of the temperature monitoring. An overview of the AP system setup is shown in Figure 3.

### 2.8 Assessment of fetal health on the AP circuit

#### 2.8.1 Physiological monitoring

Circuit flow and HR were measured with an HXL tubing flow sensor (Transonic Systems Inc.) and continuously recorded using LabChart 8 (ADInstruments Inc.). Data were sampled at a rate of 1000 Hz. At the completion of the study, data were extracted in consecutive 30‐s epochs.

#### 2.8.2 Blood gas measurements and sampling regime

UV blood gas measurements were obtained every hour including pH, PCO_2_, PO_2_, BE, bicarbonate (HCO_3_), SO_2_, and CO_2_ content (TCO_2_). In addition, sodium (Na^+^), potassium (K^+^), ionized calcium (Ca^++^), glucose, Hct, lactate, Hgb, and activated clotting time (ACT) kaolin were measured using a blood analyzer (iSTAT1; Abbott Point of Care, Inc).

#### 2.8.3 Imaging

A comprehensive fetal echocardiogram was performed by a fetal radiologist on the AP (*n* = 1) using the Vivid S6 machine (GE Healthcare). The echocardiographic assessment included the measurement of cardiothoracic ratio, tricuspid valve annulus, ductus arteriosus patency and cine short‐axis 2D imaging and M‐mode for ventricular function and Doppler patterns in the UA, UV, and ductus venosus (DV). Ultrasound was also used to examine subjects for the presence of fluid collections in the pericardial and peritoneal spaces and confirm patency of the ductus arteriosus in all of the 12 successful trials.

### 2.9 Pharmacological intervention on the AP circuit

During transition from maternal/uterine circulation to the AP, fetuses were empirically dosed once with 5 μg/kg epinephrine (Pfizer Canada Inc.), 80 mg/kg calcium chloride (Omega Laboratories LTD.), and 100 units/kg UFH (100 µg/kg) delivered via the circuit. These medications were then given individually as needed based on physiological and laboratory parameters with the goal of maintaining ACT > 300 s, Ca^++^ levels >1.4 mmol, circuit flows >80 ml/min and fetal HR > 140 beats per minute (bpm). Once circuit flows were established, IV fluids were infused via the circuit to provide a total fluid intake of 4–8 ml/kg/h. Infused maintenance crystalloid fluids (NS, PlasmaLyte) contained dextrose 6 mg/kg/min (D50; Pfizer Canada Inc.), 0.1 μg/kg/min prostaglandin E1 (PGE_1_; Pfizer Canada Inc.) and 100 units/kg/h UFH. PGE_1_ was infused to maintain patency of the fetal ductus arteriosus (Usuda et al., 2017). UFH was delivered to maintain a fetal blood ACT > 300 s to prevent thrombosis in the circuit. Dextrose 10% was provided for fetal energy requirements. For the last 9/27 experiments, 2 mg/kg hydrocortisone (Pfizer Canada Inc.) and 100 mg/kg piperacillin/tazobactam (Sandoz Inc.) were given via the circuit every 6 and 8 h, respectively. Hydrocortisone was given for hemodynamic support, while piperacillin/tazobactam was used as a broad‐spectrum empirical antibiotic. The oxygen blender (OxyDial; STARR, Life Science Corp.) supplied the oxygenator with a mixture of medical air, oxygen, and nitrogen, initially titrated to achieve UV PO_2_ of 60–80 mm Hg and PCO_2_ of 50–60 mmH g. Maternal blood was added to the circuit if Hgb concentrations fell below 100 g/L. Fetal pigs received a dose of 106 mg/kg Euthanyl after the completion of the AP experiment or if fetal distress was detected and the experiment was ended prematurely.

### 2.10 Statistics

The effect of gestational age on fetal blood gas and biochemical values was determined using a one‐way ANOVA with Bonferroni's correction for multiple comparisons. Changes in UV blood flow, HR, and temperature were analyzed using a repeated measures one‐way ANOVA with a Bonferroni correction for multiple comparisons. Blood gases, electrolytes, lactate, and glucose in utero were compared with those obtained on the AP using a Mann–Whitney Students *t* test. Sow 7 and 27 were excluded from all statistical analyses because the UV flow data for these two animals decreased rapidly and continuously from the beginning of data recording until demise.

*p *< .05 were considered statistically significant. All statistical analyses were performed using GraphPad Prism 8. The data are presented as mean ± standard deviation (SD), unless otherwise stated.

## 3 RESULTS

Twelve fetal piglets with a gestational age of 98 ± 4 days were successfully maintained using AP support for 684 ± 790 min (range 195–3,077 min) with an average body weight of 743 ± 350 g at the end of the experiment (Table 1). The Maquet Quadrox‐ID pediatric and the Maquet Quadrox‐I neonatal were the two most frequently used membrane oxygenators in our successful AP trials, although the type of oxygenator used did not bear any relation to the outcome of the experiments (data not shown). Mean UV flow normalized to body weight on AP support was 97 ± 39 ml/min/kg, which was significantly lower than the in utero UV flow of 173 ± 45 ml/min/kg (*p *= .002) by MRI. Mean fetal HR on the AP was significantly higher than in utero (206 ± 38 vs. 130 ± 10 bpm; *p *= .0007; Table 2).

**TABLE 2:** Comparison of cardiovascular parameters in utero and on AP support

	In utero		On AP	*p* value
Technique	Catheter	MRI	Flow probe	
Gestational age (days)	106 ± 0	107 ± 3	98 ± 4	NA
Weight (kg)	1.26 ± 0.15	0.62 ± 0.10	0.74 ± 0.35 (*n* = 7)	NA
Sample size	Sows (*n* = 6); Fetuses (*n* = 6)	Sows (*n* = 4); Fetuses (*n* = 20)	Sows (*n* = 10); Fetuses (*n* = 10)	NA
UV flow (ml/min/kg)	NA	173 ± 45 (*n* = 16)	97 ± 39 (*n* = 5)	**.002**
Heart rate (bpm)	130 ± 10	NA	206 ± 38 (*n* = 8)	**.0007**
Mean arterial pressure (mmHg)	37 ± 9	NA	NA	NA

Note

Mann–Whitney *U* test, Student's *t* test.

Abbreviation: NA, not available.

Bold indicates statistical significant value.

### 3.1 Hemodynamic assessment of the Yucatan mini pig while supported on the AP circuit

**FIGURE 4:**
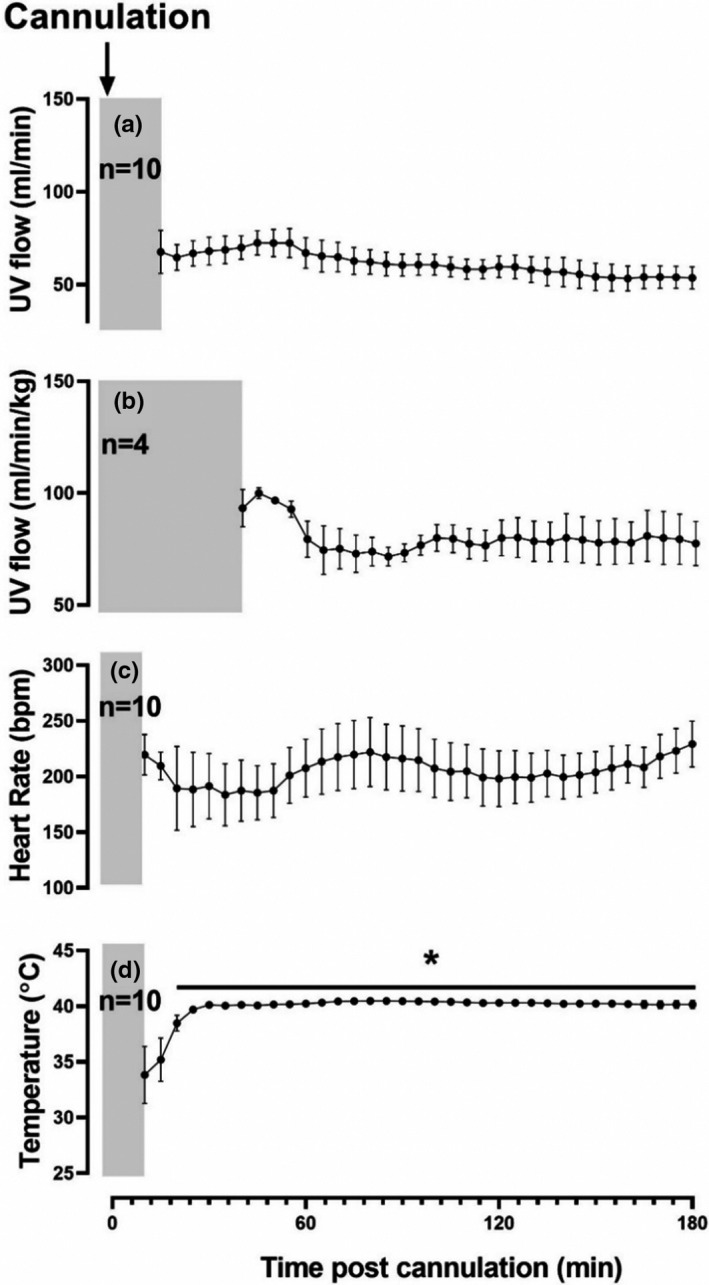
Changes in hemodynamic parameters within the first 180 min of AP support. Mean UV flow (a); UV flow normalized to body weight (b); fetal HR (c); and temperature in the BioBag over the first 3 h of AP support (d). Data from 2 of the 12 animals have been excluded from the mean data because their UV flow decreased rapidly and continuously from the beginning of recording data to demise. Their UV flow data are shown in an individual graph in Figure 6 (Figure 6b‐h, and j‐l). All values are expressed as mean ± SD and plotted when *n* > 2. Data were analyzed by a repeated measures one‐way ANOVA with a Bonferroni correction. *p < .05 statistically significant from first recorded data point. Grey bar represents the time to cannulation, connection to the flow meter, and placement in the BioBag

**FIGURE 5:**
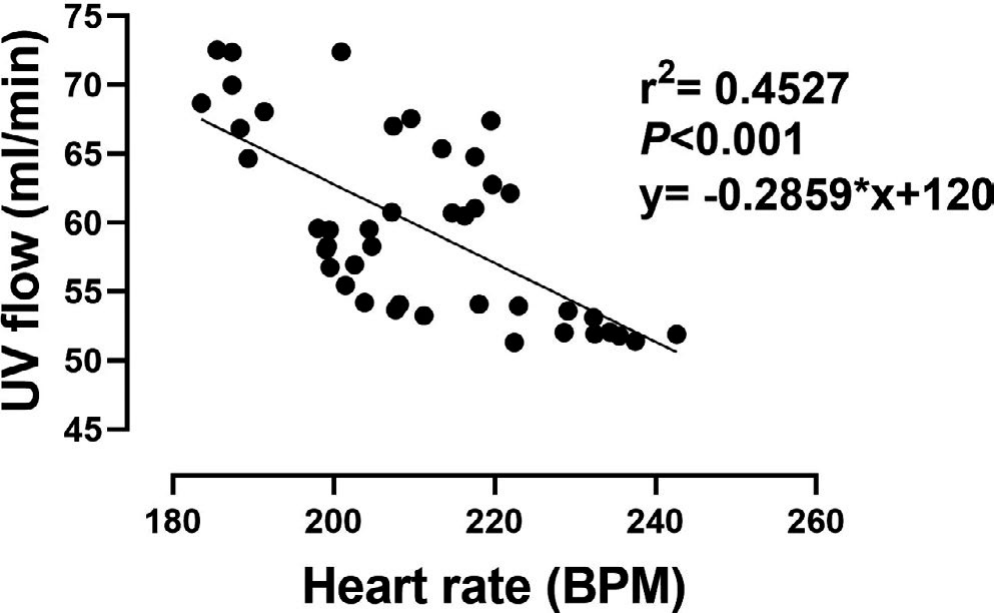
Relationship between mean heart rate and UV blood flow during the first 3 h on the AP circuit. There is a negative correlation between HR and UV flow. HR on the AP circuit were significantly higher than in utero (*p = *.0007). Each data point represents the mean of a 5‐min epoch during the first 3 h of AP support

Neither UV flow (*n* = 10) nor UV flow normalized to body weight (*n* = 4) changed during the first 180 min post‐cannulation (Figure 4a,b). Similarly, fetal HR did not change significantly over the first 180 min of AP support (Figure 4c). We observed a negative correlation between HR and UV flow (Figure 5; *r* ^2^ = .4527; *p* < .001). Approximately 20 min after cannulation, the temperature of the BioBag plateaued and remained constant throughout the first 180 min of AP support, although this represented a significant increase from the first recorded data point (Figure 4d).

**FIGURE 6:**
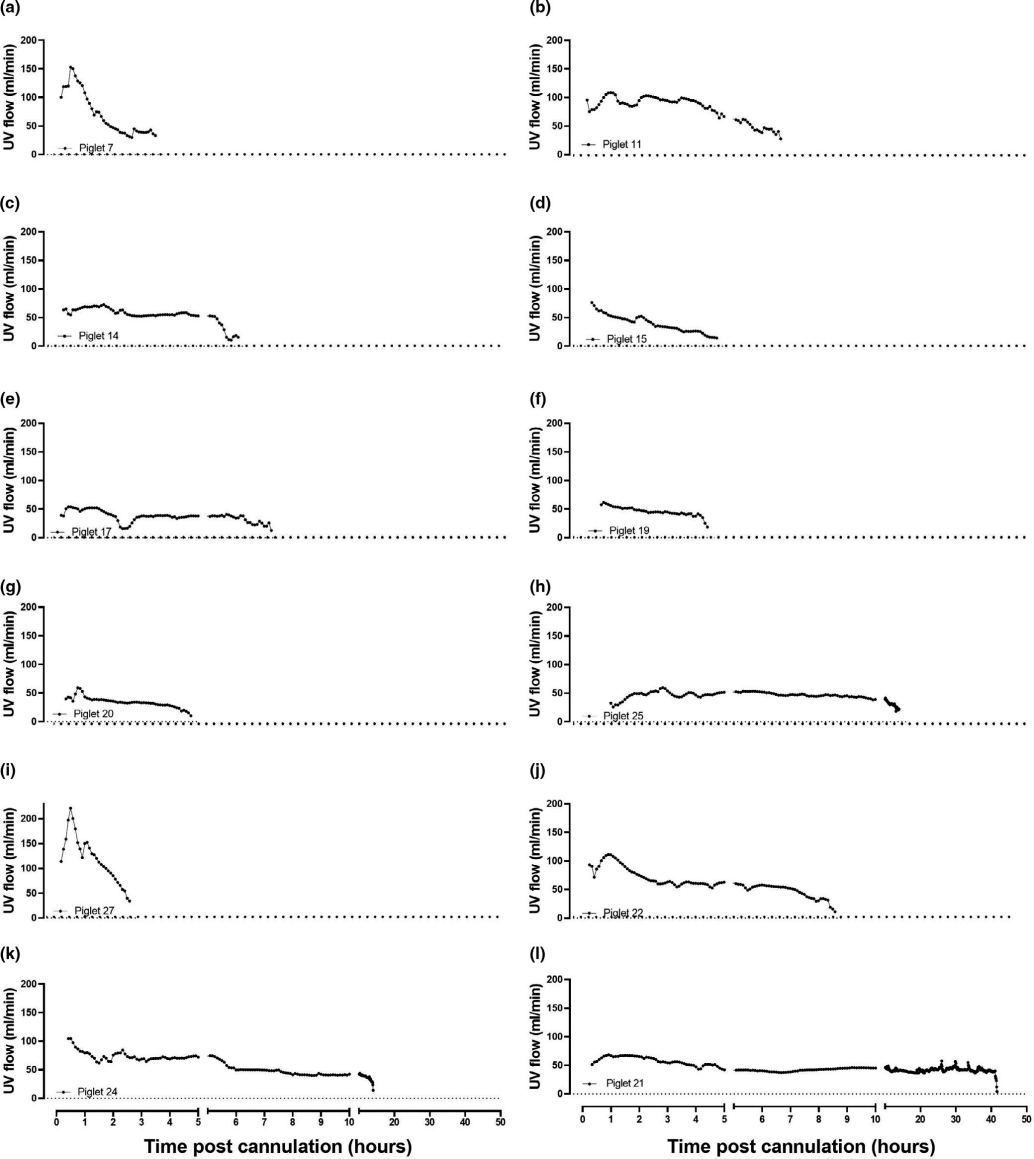
Individual UV blood flow data for each fetal piglet for the duration of AP support. All values are expressed as the mean of every 5‐min epoch for the duration of each piglet's time on the AP circuit

The 12 piglets that were successfully transitioned to the AP demonstrated different patterns of UV flow over the experimental period. In the longer AP experiments (>6 h of support), mean UV flow remained relatively stable, with a gradual decrease in UV flow over time (Figure 6b‐f,h,j‐l). In the fetuses that survived for a shorter period on the AP, UV flow was less stable. In two experiments, UV flow increased rapidly but was followed by a terminal decline thereafter (Figure 6a,i).

### 3.2 Blood gase*s*, electrolytes, and biochemical assessment in utero and on the AP circuit

**FIGURE 7:**
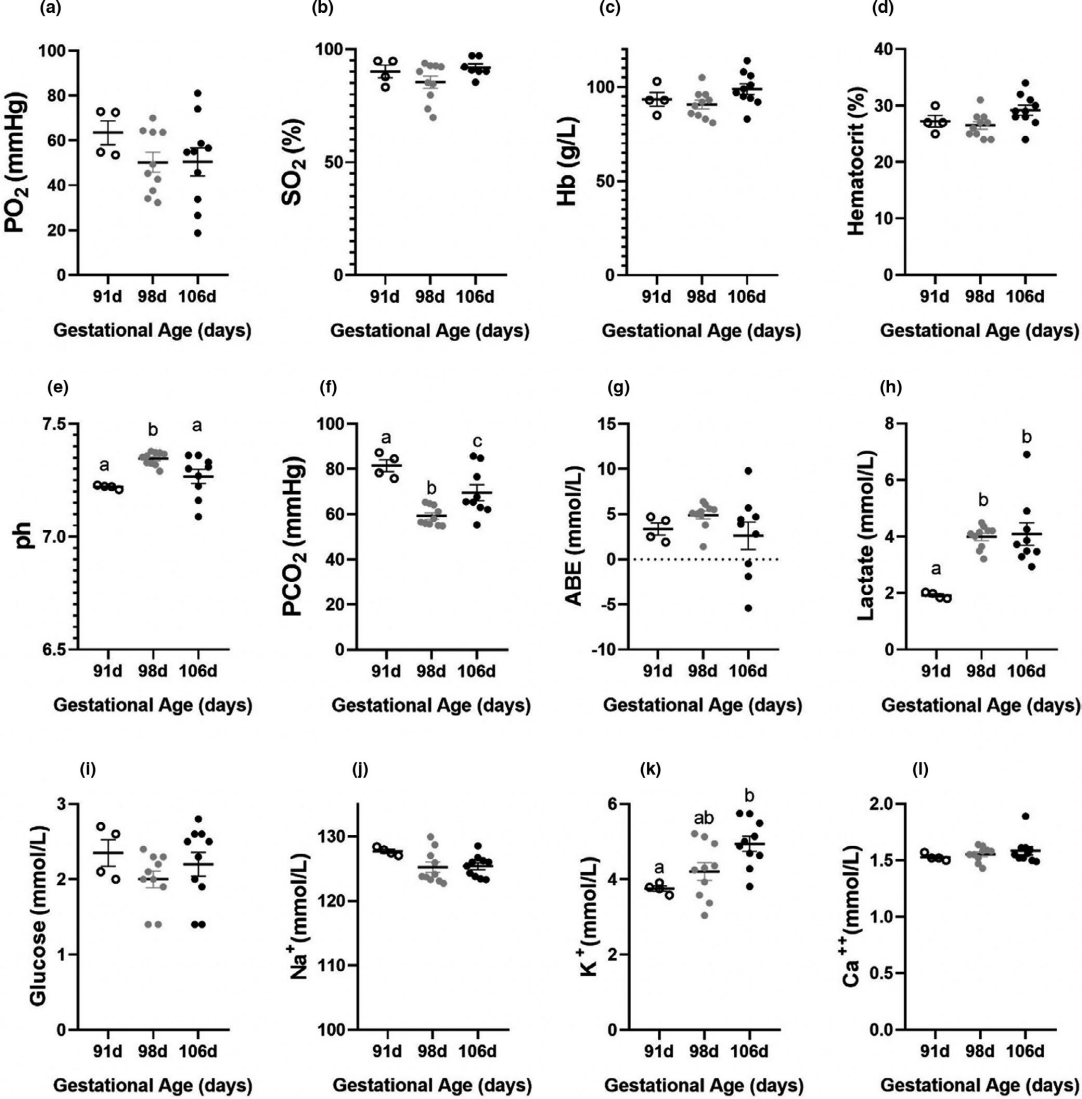
In utero blood gases, electrolytes, and biochemical values across a range of gestational ages. In utero blood gas and electrolyte values at 91 days (open circles), 98 days (grey circles), and 106 days (black circles) gestation in the UV (a‐l). Data were analyzed by a one‐way ANOVA with a Bonferroni correction for multiple comparisons. Differences between gestational ages are denoted by different letters, for example, 91 and 98 days are different in panel e as denoted by a and b; *p < *.05

**FIGURE 8:**
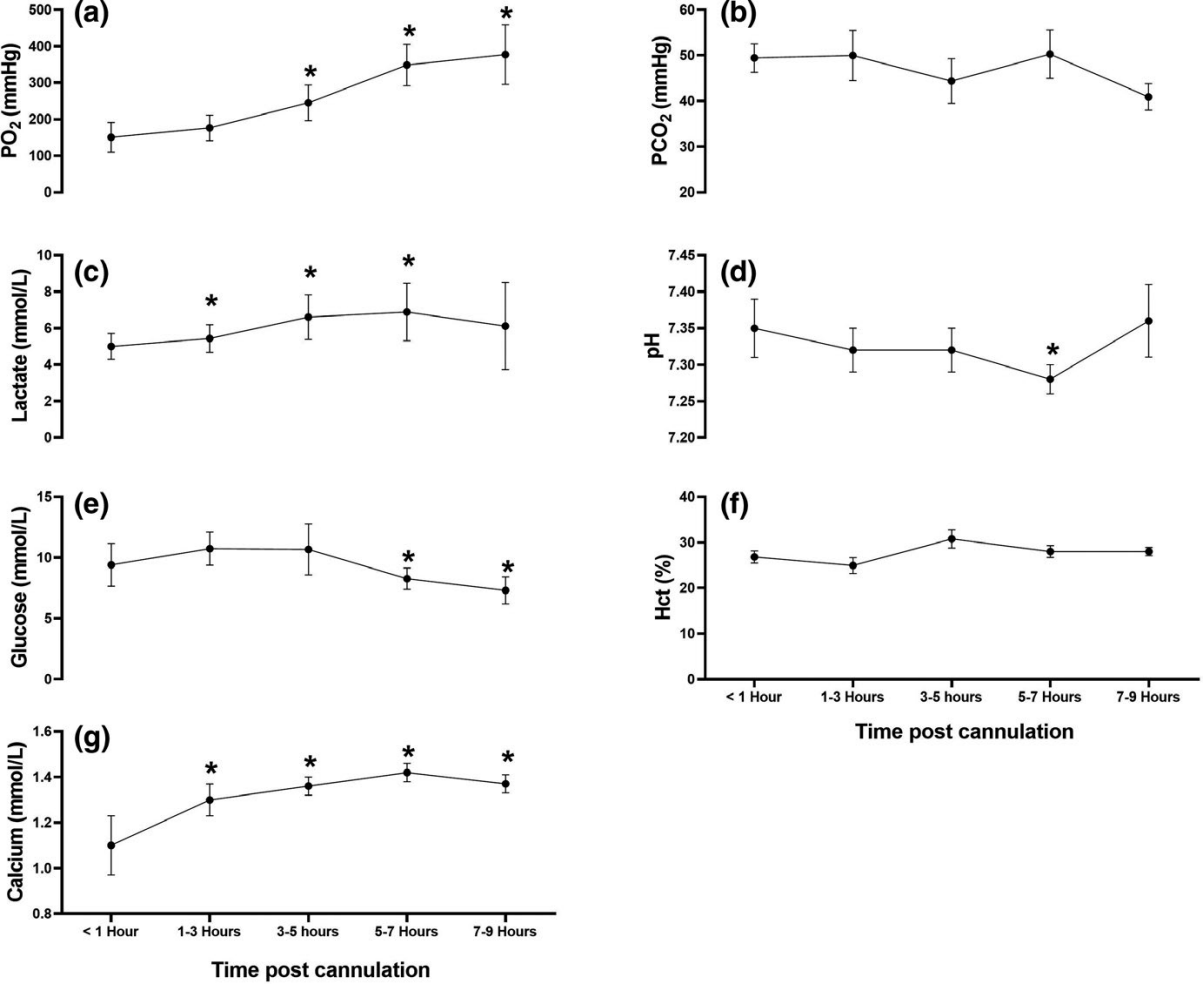
Variations in blood gases, electrolytes, and biochemical parameters over time on the AP. Changes in blood gases, lactate, glucose, and electrolyte measurements when sampled from the UV at: <1, 1–3, 3–5, 5–7, and 7–9 h, of AP support. Data were analyzed by one‐way ANOVA with Bonferroni correction for multiple comparisons; *p < .05 statistically different to first recorded data point (< 1‐h sample)

We sampled UV blood at 91, 98, and 106 days gestation to assess changes in blood gases, electrolytes, lactate, and glucose concentrations in utero across a range of gestational ages and improve the validity of our comparison of in utero results to the animals that were successfully cannulated and supported on the AP. There was no significant difference between in utero UV PO_2_, SO_2,_ Hgb, Hct, BE, glucose, Na^+^, and Ca^++^ across this gestational age range in fetuses studied in utero (Figure 7). There was no significant difference in pH at 91 and 106 days gestation. However, at 98 days gestation, pH increased significantly compared to 91 and 106 days gestation. PCO_2_ was significantly different at each gestational age. Lactate concentrations at 91 days gestation were significantly lower than at 98 and 106 days gestation, whereas there was no significant difference in lactate concentrations at 98 and 106 days gestation. K^+^ at 106 days gestation was significantly greater than K^+^ at 91 days, while there was no significant difference in K^+^ concentrations at 91 and 106 days gestation compared to 98 days gestational age (Figure 7). PO_2_, glucose, and Na^+^ were significantly greater on AP support versus in utero whereas, PCO_2_, BE, and Ca^++^ were significantly lower on the AP compared to in utero UV measurements (Table 3). However, there was no significant difference in Hgb, pH, Hct, lactate, and K^+^ between the animals on AP support compared to in utero animals (Table 3). After 3–5 h of AP support, PO_2_ increased significantly. Lactate concentrations increased significantly after the first hour of support but returned to baseline at 7–9 h. pH remained relatively constant during AP support, except at 5–7 h when it decreased. This decrease in pH coincided with an increase in lactate at 5–7 h post‐cannulation. Glucose concentration remained relatively stable across AP support but decreased significantly at 5–7 h. Ca^++^ increased significantly at all sample times, which may have reflected Ca^++^ supplementation throughout the experiment, although it remained below in utero concentrations throughout (Figure 8 and Table 3). There was no significant difference in Hct or PCO_2_ throughout AP support (Figure 8).

**TABLE 3:** Comparison of UV blood gases, lactate, glucose, and electrolyte measurements in utero and within the first 2 h of AP support in fetal piglets at 91–106 days gestation. In the AP group, only 9 of the 12 piglets successfully transitioned onto the AP were included in this comparison

	In Utero (*n* = 24)	AP (*n* = 9)	*p* value
PO_2_ (mm Hg)	53 ± 16	280 ± 176	**.0029**
PCO_2_ (mm Hg)	67.1 ± 11.0 (*n* = 23)	54.9 ± 11.7	**.0165**
pH	7.293 ± 0.0768 (*n* = 23)	7.298 ± 0.0643	.5431
Hemoglobin (g/L)	95 ± 9	88 ± 20 (*n* = 8)	.7406
SO_2_ (%)	83 ± 16	86 ± 19	.0995
BE (mmol/L)	3.7 ± 3.1 (*n* = 23)	0.1 ± 4.3	**.0236**
Hematocrit (%)	28 ± 3	26 ± 6 (*n* = 8)	.9576
Glucose (mmol/L)	2.1 ± 0.4	9.8 ± 5.9 (*n* = 8)	**<.0001**
Lactate (mmol/L)	3.98 ± 1.88	4.23 ± 2.38 (*n* = 7)	.4346
Na^+^ (mmol/L)	126 ± 2	133 ± 3 (*n* = 8)	**<.0001**
K^+^ (mmol/L)	4.4 ± 0.8	5.3 ± 1.5 (*n* = 8)	.2157
Ca^++^ (mmol/L)	1.56 ± 0.087	1.29 ± 0.21 (*n* = 7)	**.0029**
Cl^−^ (mmol/L)	96 ± 2	NA	NA

Note

Mann–Whitney *U* test, Students *t* test.

Abbreviations: NA, not available.

Bold indicates statistical significant value.

### 3.3 Echocardiographic assessment over time on the AP circuit

**FIGURE 9:**
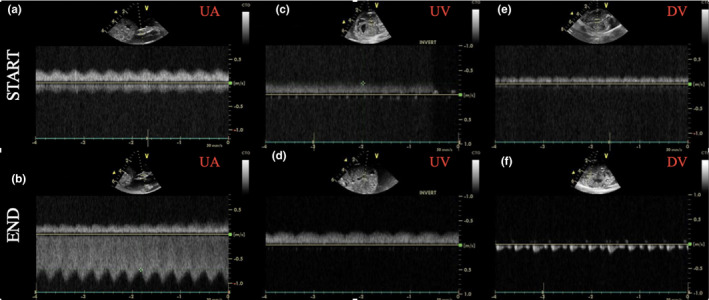
Pulsed Dopplers of the UA, UV and DV at the start and end of AP support. Example image of pulsed Dopplers of the UA (a,b), UV (c,d), and DV (e,f) at the start (a,c,e) and at the end (b,d,f) of AP support. At the start (within the first hour) of AP support UA, UV, and DV velocity waveforms were normal (a,c,e); however, toward the end (within the last 2 h) of the AP support waveform patterns became abnormal (b,d,f)

**FIGURE 10:**
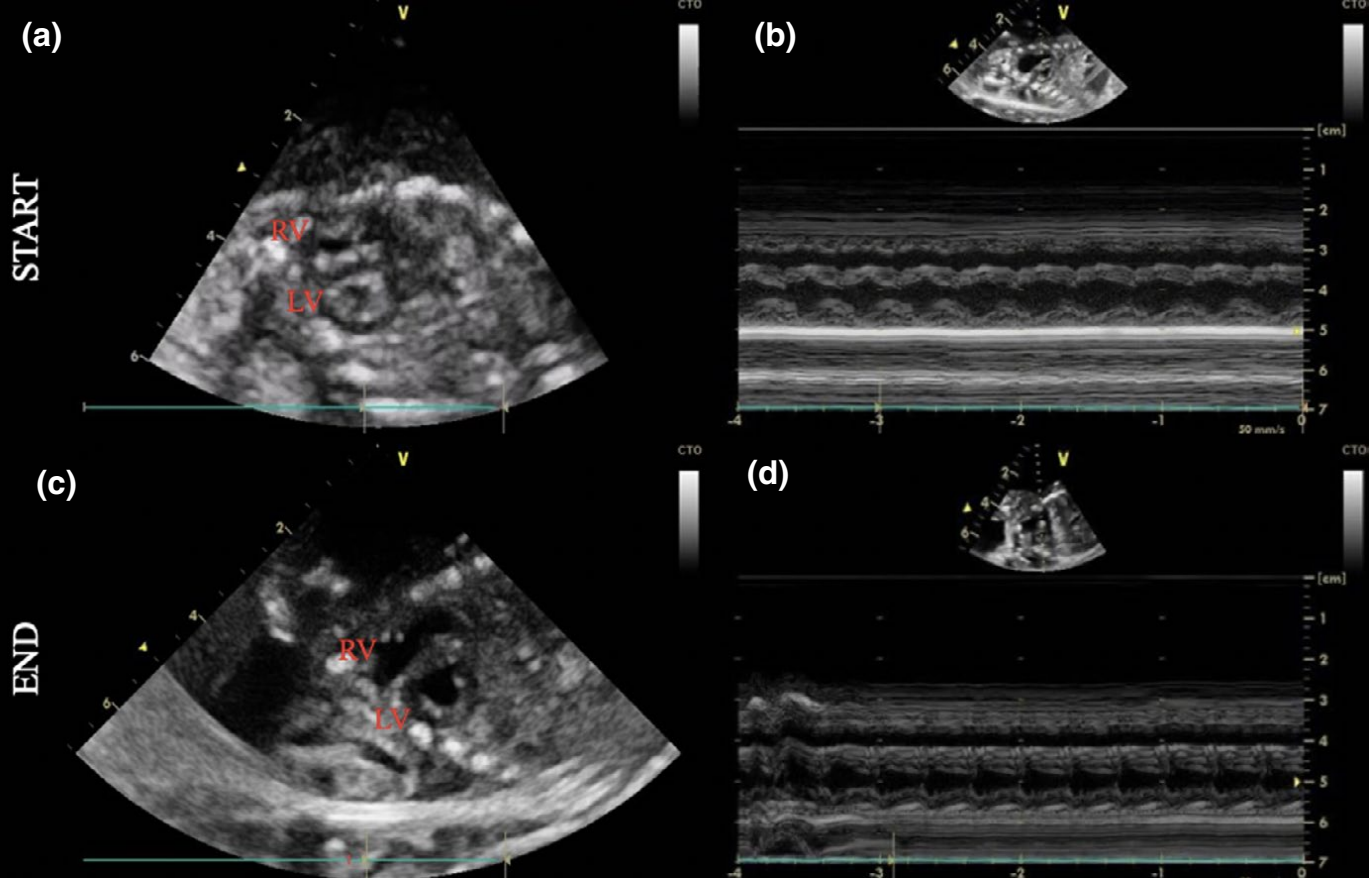
Fetal echocardiography at the start and end of the time on the AP circuit. Fetal echocardiography showing short‐axis view (a,c) and M‐mode (b,d) at the start (a,b) and end (c,d) of AP support. At the start of AP support, RV morphology and function were normal (a,b); however, at the end of AP support shows RV enlargement and diminished contractility (c,d)

Ultrasound scans confirmed the presence of ascites and pericardial effusions in keeping with hydrops in all of our subjects. We confirmed the patency of the ductus arteriosus in all subjects and performed a more comprehensive serial echocardiographic assessment of a single animal on the AP. Echocardiography was performed every ~3 h on this piglet over 917 min of AP support (Table 4). UA pulsatility index, cardiothoracic ratio, tricuspid valve size, ascites, and pericardial effusion increased progressively throughout the duration of AP support. There was no difference in left ventricular ejection fraction from the start of AP support to demise (Table 4). Normal pulsed Dopplers of the UA, UV, and DV were observed at the start of AP support, but as the duration of AP support progressed, we observed velocity and pulsatility index changes in UA, pulsatile flow in UV (decreased UV velocity during atrial systole) and absence or reversal of the DV a‐wave (Figure 9). Similarly, short‐axis and M‐mode views of the right ventricle (RV) demonstrated progressive enlargement and reduced contractility of the RV on the AP circuit (Figure 10 and Videos S1 and S2).

**TABLE 4:** Changes in echocardiography parameters, and pulsed Doppler indices when assessed every 2.5 h over 917 min of AP support in a single fetus (Piglet 25, Table 1)

Time (h)	UA (PI)	UV	DV	CTR	LV EF (%)	TV size (mm)	Ascites (mm)	Pericardial effusion (mm)
2.5	0.39	(−)	(−)	0.28	53	6.70	2	0
5	0.40	(−)	(−)	0.42	62	7.70	2	0
7.5	0.43	(+)	(+)	0.42	72	8.70	5	0
10	0.62	(+)	(+)	0.43	56	9.80	8	0
12.5	0.54	(+)	(+)	0.63	66	9.20	12	2
15	NA	NA	NA	NA	NA	NA	18	2
Mean ± SD	0.48 ± 0.10	NA	NA	0.44 ± 0.12	62 ± 7.6	8.42 ± 1.23	8 ± 6	NA

Abbreviations: (−), normal; (+), abnormal;CTR, cardiothoracic ratio; DV, ductus venosus; LV EF, left ventricle ejection fraction; NA, not available; PI, pulsatility index; SD, standard deviation; TV, tricuspid valve; UA, umbilical artery; UV, umbilical vein.

## 4 DISCUSSION

In this study, we investigated the feasibility of a short‐term model of AP support of the preterm miniature pig using a commercial neonatal or pediatric hollow fiber membrane oxygenator connected to the fetal circulation via the extra‐abdominal umbilical vessels, while incubating the fetus in an artificial uterine environment. We successfully cannulated twelve fetal pigs onto the AP for 684 ± 790 min with all experiments ending with progressive cardiovascular deterioration and hemodynamic instability. Our findings indicate that (1) AP support of the preterm miniature pig at 98 ± 4 days is feasible; (2) circuit flow is subphysiologic in animals supported with the same commercial neonatal oxygenators and UA and UV cannulas that have been associated with normal umbilical flows in larger fetal sheep; and (3) subphysiologic UV flows are associated with the development of signs of heart failure including tachycardia, elevated central venous pressure, right ventricular dysfunction and hydrops fetalis.

While there are many advantages of using a fetal sheep model to investigate AP technology, there are several questions that remain with regard to the feasibility of translating these results to human subjects. One difference between sheep and humans is with respect to the umbilical cord anatomy. As previously discussed, the sheep umbilical cord contains two UAs and two UVs in contrast to humans and pigs, who generally have one UV and two UAs (Benirschke & Kaufmann, 2000; Steven, 1968). The presence of two pairs of umbilical vessels may facilitate umbilical vessel cannulation and transition of the fetal sheep onto the AP by allowing the cannulation of one pair of UA and vein, while the other pair of vessels‐maintained circulation through the native placenta. In contrast, to prevent fetal hypoxemia and disruption of fetal circulation in both human and pig, the connection of a three‐vessel cord to the oxygenator will require timely cannulation of smaller umbilical vessels, with cannulas that need to be as large as possible in relation to the umbilical vessel they are connected to. It is hoped that our experience with a three‐vessel cord may, therefore, be helpful for the translation of the technique to human subjects.

From a developmental perspective, lung maturation in the fetal sheep at 105–116 days gestation is analogous to the human fetus at 23–25 weeks gestation (Miura et al., 2016; Partridge et al., 2017; Partridge, Davey, Hornick, McGovern, et al., 2017). However, at this gestational age, fetal sheep weigh approximately 1.0–2.5 kg, which is significantly more than a human fetus at 23–25 weeks gestation (Hornick et al., 2018; Partridge, Davey, Hornick, & Flake, 2017). Usuda et al. reported success with AP support of fetal sheep at 95 days gestation weighing 600–700 g using a customized low volume oxygenator (Usuda et al., 2019). In addition, in those experiments, large doses of corticosteroids and continuous infusions of phosphodiesterase inhibitors were required to overcome a period of “refractory hypotension” and “improve organ perfusion” following initiation onto the circuit. The weight of our subjects was 743 ± 350 g (post‐AP support) at 98 ± 4 days gestation, which is comparable to the human fetus at 26 weeks. Ours and others’ experience would suggest that a fetal mini pig born a few days earlier (91–94 days gestation) would be the same size with equivalent lung developmental stage as a 23–25 weeks human, further supporting the appropriateness of the fetal pig as a translatable model of AP support of the preterm human (Eiby et al., 2013).

Compared with in utero controls, our AP experiments were characterized by subphysiologic UV flow and tachycardia, and although we did not perform routine echocardiographic assessments of all piglets successfully cannulated onto the AP, our results from a single experiment demonstrate a progressive increase in the cardiothoracic ratio, tricuspid valve size, ascites, and pericardial effusion over 917 min of AP support (Table 4). M‐mode and short‐axis echocardiographic assessment of the RV demonstrated enlargement and diminished contractility of the RV, suggesting the development of right heart failure over the duration of AP support (Figure 10). We speculate that the changes in RV function were induced by excessive RV afterload caused by (1) supraphysiologic circuit resistance (Miura et al., 2012; Reoma et al., 2009); and (2) excessive total peripheral resistance caused by a heightened sympathetic nervous system (SNS) in response to low UV flow and the consequent impairment of venous return.

We suspect that the small size and weight of our preterm minipigs were associated with smaller umbilical vessel diameters than those present in the previously described fetal sheep models of the AP (Köşüş et al., 2012). Umbilical vessel cannulation further reduces the diameter and area of the vessel by the wall thickness of the cannula, which becomes more significant as vessel size and distensibility decreases. According to Poiseuille's law, resistance is inversely proportional to the fourth power of the radius and directly proportional to the vessel length and the viscosity of the fluid. By cannulating the umbilical vessels, the smallest internal radius is defined by the inner diameter of the cannula, and thus resistance within the circuit increases (Broman et al., 2019; Gordan et al., 2015). Additionally, it is possible that the relatively larger priming volume in relation to total fetal circulating volume further contributes to energy loss in the extracorporeal circuit compared to larger animals. In sheep, the proportion of the fetal circulating volume within the umbilical cord and placenta drops from >50% at half gestation to <20% at term, and it is possible that total indexed fetal circulating volume declines over gestation. Based on measurements of the volume of blood within the fetus of 80 ml/kg, the blood volume within the fetal side of the placenta at mid‐gestation is likely to be less than 80 ml/kg of fetal weight (Kiserud & Acharya, 2004), translating to approximately 40 ml for a 500 g fetus. During the cannulation of the umbilical vessels, we struggled initially to maintain arterial patency due to vasospasm of the UAs. We believe that this may have also contributed to increased resistance in the circuit, imposing additional afterload on the RV. Efforts to prevent cord spasm with topical papaverine and warmed saline significantly improved the cannulation procedure; however, this did not prevent cord spasm after umbilical vessel cannulation.

In our successful attempts to provide AP support to the preterm minipig, UV flow was significantly lower than the flow we observed in utero using our established MRI methods validated in human and sheep fetuses (Duan et al., 2019; Jansz et al., 2010; Seed et al., 2012). We speculate that this relationship may be attributable to increased circuit resistance. In accordance with Ohm's law, flow is dependent on the pressure gradient across the circuit and inversely related to the resistance. Therefore, increasing the resistance leads to a reduction in circuit flow (Hornick et al., 2018). Additionally, at 106 days gestation MAP in the preterm piglet (37 ± 9 mm Hg) is lower than in larger preterm sheep (47 ± 5 mm Hg) previously supported on the AP (Coughlin et al., 2019), indicating that there may be insufficient arterial pressure to maintain normal UV flow in our experiment (Table 2). With declining circuit flow, it becomes increasingly difficult to achieve adequate oxygenation, often resulting in reduced fetal oxygen delivery, metabolic acidosis, and circulatory collapse (Carter, 1989; Omo‐Aghoja, 2014; Rudolph, 2009). To compensate for low UV flow and to sustain adequate fetal oxygen delivery, we intentionally increased PO_2_ and SO_2_ by increasing the oxygen sweep to the post‐membrane side of the oxygenator, which accounts for the supraphysiologic PO_2_ and SO_2_ we observed in the AP group compared to the in utero animals (Table 3, Figure 8).

The fetal minipigs that were successfully cannulated onto the AP were tachycardic compared to the in utero group (Table 2). Moreover, we observed an inverse correlation between fetal HR and UV flow on the AP (Figure 5). We speculate that the increase in fetal HR is evidence of sympathetic response to reduced umbilical venous return, effectively shifting the balance between systemic and artificial placental perfusion. This results in a shift of the pressure‐volume relationship toward lower stroke volume (SV) due to the higher afterload and, higher end‐diastolic filling pressure, but also higher stroke work. This initiates an increase in sympathetic tone and peripheral vasoconstriction that imposes further afterload on the RV as already well‐described for heart failure (Danielson et al., 2005; Jones et al., 1987; Rudolph, 2009; Ruijtenbeek et al., 2002; Schuijers et al., 1986; Yiallourou et al., 2013). It has been shown previously that the fetal heart is particularly sensitive to high afterload (Kamitomo et al., 1992). A telling example of how the fetal heart responds to an acute increase in RV afterload is in the setting of ductus arteriosus (DA) occlusion. Acute occlusion of the DA in fetal sheep increases afterload and induces RV chamber enlargement and hypertrophy, with reduced fractional shortening (Tulzer et al., 1991). Our echocardiography data at the start of AP support demonstrate patency of the DA and no flow acceleration (Video S3). Therefore, premature closure of the DA is an unlikely explanation for the observed heart failure on the AP circuit.

There are several limitations in the current study. First, our comparison of hemodynamic, blood gas, and biochemical parameters *in utero*, and on the AP was performed in two different breeds of pigs. There are likely some differences, specifically a significant difference in maternal and litter size that may have ultimately affected the interpretation of our study results. Another limitation of the current study was our lack of invasive central venous pressure, systemic and umbilical arterial pressure, urine output measurements, biochemical markers of SNS activity, circuit resistance, and myocardial injury. Consequently, we have not fully characterized the underlying mechanisms driving the subphysiologic UV flow and the development of right heart failure on the AP circuit. We suggest these hemodynamic parameters should be monitored in future experiments to better understand fetal hemodynamics on the AP circuit. Unfortunately, we collected limited echocardiographic data in this pilot study, and future studies will expand on this to inform data interpretation. The mean weight of the animals at the end of AP support was 743 ± 350 g, which was likely significantly greater than the animals’ weights at the start of the trial as the presence of hydrops likely resulted in significant weight gain on the AP circuit. This is supported by the fetal weight calculated by MRI (0.62 ± 0.11 kg; Table 2), which was lower than the fetuses previously supported on the AP, despite being scanned at a later gestational age (107 ± 3 vs. 98 ± 4 days; Table 2).

## 5 FUTURE DIRECTIONS

Recent breakthroughs in the application of AP technology have employed pumpless extracorporeal oxygenator circuits (Partridge, Davey, Hornick, McGovern, et al., 2017; Usuda et al., 2019). We note that hemodynamic stability in these experiments was achieved with low volume oxygenators with membranes suitable for long‐term use that are not available on a commercial basis. We would suggest that even smaller oxygenators may be needed for pumpless systems suitable for use in extremely preterm human infants (Schoberer et al., 2014). An alternate approach to overcoming excessive resistance and maintaining physiologic circuit flows might be to introduce mechanical support to the circuit. Early successes with AP technology incorporated pumps in the circuits, although none achieved the degree of circulatory stability reported in recent pumpless systems (Westin et al., 1958; Zapol et al., 1969). However, presumably, as vessel diameters and arterio‐venous blood pressure gradients diminish in tandem with lower weights, the feasibility of maintaining normal umbilical flow in a pumpless circuit becomes more uncertain. The recent advent of highly successful extra‐corporeal membrane oxygenation circuit designs for neonates that are powered by small centrifugal pumps raises the possibility of incorporation of a miniaturized pump into an artificial placenta circuit designed for extremely preterm infants.

## 6 CONCLUSION

At ~95 days gestation, the miniature pig fetus represents a realistic model of the extremely preterm human infant that can help facilitate the clinical translation of AP technology. Although we have demonstrated the feasibility of initiating this form of support in a premature miniature pig model, we were unable to achieve stable hemodynamics, which we attribute to excessive resistance in the circuit. We, therefore, conclude that a pumpless AP circuit based on a commercially available neonatal or pediatric oxygenator is unlikely to represent a feasible approach for supporting a 500 g fetus. However, if a modification in circuit design can more realistically simulate the circulatory physiology of the placenta through the miniaturization of the oxygenator and/or the addition of mechanical support, it may be possible to achieve the same hemodynamic stability that has previously been reported in larger fetal sheep. The technical challenges involved in cannulating the umbilical cord vessels, including significant problems with cord vessel spasm, as well as potential issues with infection, inflammation, and thrombosis during longer runs may be significant barriers to clinical translation. However, given the relative plateau of outcomes in babies born at the lower limit of viability seen over recent years, the appeal of this approach to providing a more physiologically appropriate form of intensive care to the extremely preterm infant remains compelling.

## 7 TRANSLATIONAL PERSPECTIVES

Respiratory failure is arguably the greatest challenge for the extremely preterm infant to overcome due to severe immaturity of the lungs. At the lower limit of viability, the lungs lack the necessary alveolar development required to support the transition from placental to pulmonary gas exchange. Efforts to support pulmonary development with mechanical ventilation are harmful to the immature lungs causing irreversible lung injury and pulmonary developmental arrest. Studies using preterm fetal sheep show that artificial placenta support promotes lung development and protects the immature lungs from injury when compared to mechanical ventilation. Additionally, white‐matter injury in preterm sheep was avoided on artificial placenta support and neurodevelopment and cortical folding occurred normally and in parallel with that of in utero brain development. Despite great success with artificial placenta support in preterm sheep, this model may not accurately reflect the weight and umbilical cord anatomy of the preterm human, highlighting the need for other animal models that bring this technology closer to the clinical translation. In contrast to sheep, the preterm pig shares similar umbilical cord anatomy and weight to the preterm human neonate and represents realistic challenges of umbilical vessel cannulation and artificial placenta support that will be faced with translation to human subjects. We demonstrate that artificial placenta support of the preterm pig is feasible; however, challenges that were not present in artificial placenta support of the larger sheep models exist and this work brings this exciting technology closer to clinical translation.

## CONFLICT OF INTEREST

The authors have no competing interests to disclose.

## AUTHOR CONTRIBUTIONS

Conception or design of the work: MS, JLM, CH. Data acquisition: ACP, AS, LT, MJM, AF, LS, TA, SKSC, JR, LCL, CF, JL, BSS, ME, AL, JB, DM, MQ, SLH, JRTD, JLM, CH. Data analysis or interpretation: ACP, AS, LT, MJM, MQ, SLH, JRTD, MS, JLM, CH. Drafting the work or revising it critically for important intellectual content: ACP, AS, MJM, JB, JRTD, MS, JLM, CH. All authors approved the final version of the manuscript, agree to be accountable for all aspects of the work in ensuring that questions related to the accuracy or integrity of any part of the work are appropriately investigated and resolved, and all persons designated as authors qualify for authorship, and all those who qualify for authorship are listed.

## Supporting information



Video S1

Video S2

Video S3

## Data Availability

The data that support the findings of this study are available from the corresponding author upon reasonable request.
